# Post-translesion synthesis repair

**DOI:** 10.18632/oncotarget.4938

**Published:** 2015-07-21

**Authors:** Jacob G. Jansen, Anastasia Tsaalbi-Shtylik, Niels de Wind

**Affiliations:** Department of Human Genetics, Leiden University Medical Center, Leiden, The Netherlands

Unrepaired DNA nucleotide lesions, derived from endogenous (radical oxygen species, base decay, etc) or exogenous (sunlight, smoke, alcohol, etc.) sources can compromise cellular and organismal health. Cellular responses to DNA damage range from DNA damage responses (DDR) including checkpoints, senescence 1and apoptosis, to nucleotide substitutions and genomic rearrangements [1]. A common intermediate in all these responses are the lesion-containing single-stranded DNA (ssDNA) tracts that originate from the inability of replicative DNA polymerases to bypass damaged templates [2]. Persistent ssDNA tracts recruit the heterotrimeric RPA protein and the ATR/ATRIP DNA kinase that initiates DDR by phosphorylating various proteins, including the CHK1 DNA damage signaling kinase [1, 3]. When ssDNA tracts persist they are at risk to collapse into recombinogenic double-strand DNA breaks [2]. To avert the induction of DDR and of DSB, specialized DNA translesion synthesis (TLS) polymerases fill the lesion-containing ssDNA tracts. Unfortunately, TLS polymerases frequently insert an incorrect nucleotide opposite the damaged nucleotide, ultimately resulting in a nucleotide substitution. It is important, therefore, to keep TLS in check. Mechanisms that control TLS include the restricted recruitment of TLS polymerases, the selective expression or posttranslational modification of TLS polymerases or, possibly, correction of TLS errors by the proofreading activity of the replicative polymerases [3].

We recently have unveiled a new mechanism that controls mutagenic TLS, and also DDR induction, in response to physiologically relevant DNA lesion densities [4]. This mechanism utilizes MutSα, a core component of the DNA mismatch repair (MMR) pathway. During canonical MMR, MutSα recognizes and binds misincorporations by the replicative DNA polymerases opposite normal or slightly modified nucleotides. This initiates a repair cascade that involves (i) MutLα-mediated incision of the nascent DNA strand, 5′ of the mismatch, (ii) the exonucleolytic removal of the nascent DNA strand containing the misincorporation, and (iii) resynthesis [5]. Loss of MMR results in a spontaneous mutator phenotype that originates cancer in the human Lynch syndrome.

**Figure 1:**
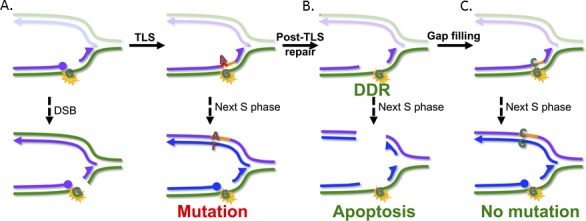
Model for the induction of DDR and the suppression of mutations by post-TLS repair Error-prone TLS at distorting nucleotide lesions (red+orange tract in panel **A**) prevents the induction of DSBs and of DDR, at the price of incorrect incorporations that result in mutations during the S phase of the next cell cycle. Post TLS repair (panel **B**) excises the TLS error. The resulting ssDNA tract induces DDR. We hypothesise that, during the next S phase, the gapped template can collapse to a DSB, mediating a delayed apoptotic response. Error-free gap filling by TLS (panel **C**, orange tract) prevents the induction of a nucleotide substitution.

It has long been known that MMR proteins additionally are involved in provoking DDR to nucleotide lesions that severely disrupt the helical structure of DNA, including polycyclic aromatic hydrocarbons and ultraviolet (UV) light [6]. It has been hypothesized that this response reflects binding of MutSα and MutLα to damaged nucleotides, followed by the direct activation of the DDR machinery [5]. However, although MutSα and MutLα can indeed recruit ATR/ATRIP and CHK1, there is no good evidence in favor of the binding of MutSα to damaged nucleotides (at least outside of the context of replication). In addition to activating DDR, MutSα plays a second role in responses to nucleotide lesions: we found that UV light is considerably more mutagenic in MutSα-deficient cells than in wild type cells. This phenotype was not caused by a requirement of MutSα for optimal nucleotide excision repair (NER), the pathway that prevents mutagenesis by excising damaged nucleotides. Surprisingly, residual DNA incision activity in UV-treated NER-deficient cells was dependent on MutSα. By staining for photolesions, embedded in ssDNA we demonstrated that MutSα directs excision of the undamaged DNA strand opposite to the photolesion. The resulting ssDNA tracts with embedded photolesions induced RPA-ATR-CHK1-dependent checkpoints. Moreover, these MutSα-dependent single-stranded photolesions most likely are converted into toxic DSBs during the S phase of the next cell cycle. Why would MutSα initiate excision of the DNA strand opposite a photolesion? As explained above, damaged nucleotides require TLS for (mutagenic) bypass. Our data therefore suggested that MutSα might direct the excision of incorrect nucleotides incorporated by TLS opposite damaged nucleotides. Indeed, whereas loss of MutSα in wild type cells resulted in a strong increase of UV-induced mutations, disruption of MutSα in TLS-defective cells led to only a marginal increase in UV-induced mutation frequencies. In genetic terms, mutagenic TLS and MutSα act in an epistatic fashion. Further evidence for the MutSα-directed excision of TLS errors came from an *in vivo* TLS assay that employs a site-specific photolesion, introduced on a replicating episome. While the absolute efficiency of TLS at the photolesion was similar in MutSα-proficient and MutSα-deficient cells, its mutagenicity was increased considerably in the absence of MutSα. In conclusion, MutSα acts to excise ‘misincorporations’ by TLS opposite non-instructive photolesions, thereby *a posteriori* controlling its mutagenicity. Consistent with our results, some evidence exists that MutSα indeed can recognize ‘mismatched’ non-instructive DNA lesions [7]. Additionally, the ssDNA tracts induced by this excision are associated with the induction of DDR and, during the S phase of the subsequent cell cycle, with DSBs and a consequent apoptotic response (Figure 1). We call this new excision repair pathway, for obvious reasons, post-TLS repair.

Currently, we do not understand how MutSα recognizes such highly aberrant, damaged nucleotide ‘mispairs’, nor do we know whether also MutLα, the mismatch repair-associated exonuclease EXO1 or other downstream proteins participate in post-TLS repair. These questions are subject to further research.
